# Clinical and functional outcomes of fracture pattern-driven plate osteosynthesis technique for comminuted patellar fractures using multiple miniplates

**DOI:** 10.1007/s00402-026-06212-8

**Published:** 2026-02-25

**Authors:** Jae-Woo Cho, Won-Tae Cho, Seungyeob Sakong, Wonseok Choi, Seonghyun Kang, Ppuri Bak, William T. Kent, Jeong-Seok Choi, Jong-Keon Oh

**Affiliations:** 1 Department of Orthopedic Surgery, Korea University Guro Hospital, Seoul, Korea, Republic of; 2 Department of Orthopedic Surgery, Ajou Univeristy Hospital, Suwon, Korea, Republic of; 3 Department of Orthopedic Surgery, University of California, San Diego, San Diego, USA; 4 Department of Orthopedic Surgery, Armed Forces Capital Hospital, Seongnam, Korea, Republic of

**Keywords:** Patellar fracture, Comminuted fracture, Plate osteosynthesis, Miniplate fixation, Fragment-specific fixation, Quadriceps strength

## Abstract

**Introduction:**

Comminuted patellar fractures, particularly AO/OTA 34-C2 and C3 fractures, present substantial challenges in achieving stable fixation and anatomical reconstruction. Conventional tension-band wiring may fail in such cases due to insufficient mechanical stability. This study aimed to evaluate the clinical and functional outcomes of a fracture pattern-driven plate osteosynthesis technique using multiple miniplates tailored to the intricate morphology of multifragmentary patellar fractures.

**Methods:**

A retrospective review was conducted of 62 patients with AO/OTA 34-C2 or C3 patellar fractures treated between 2018 and 2023 at two academic trauma centers. All patients underwent fixation using a fragment-specific approach involving anterior tension-band plating and miniplates, guided by preoperative CT-based fracture mapping. The outcome measures included radiographic reduction quality, union rate, range of motion (ROM), Lysholm score, and isokinetic quadriceps strength at 6 and 12 months.

**Results:**

Anatomical or good reduction was achieved in all cases (72.6% anatomical, 27.4% good), with a 100% union rate and low complication rate (3.2% reoperation rate). The mean final ROM was 132.9° ± 6.8°, and Lysholm scores improved from 70.3 ± 10.0 at 6 months to 89.1 ± 6.7 at 12 months. However, isokinetic peak torque deficits persisted at 12 months (mean 24.6% ± 13.0), and the body mass–normalized extension torque averaged 0.99 ± 0.40 Nm/kg, indicating residual muscle weakness despite rehabilitation.

**Conclusion:**

The fracture-pattern-driven osteosynthesis technique using multiple miniplates offers a reliable method for managing complex patellar fractures, providing excellent reduction quality, high union rates, and satisfactory functional outcomes. This approach enables individualized fixation strategies tailored to fragment morphology. Despite good clinical recovery, persistent deficits in quadriceps strength highlight the need for prolonged rehabilitation beyond 12 months to achieve complete functional restoration.

## Introduction

The ideal goals for the surgical treatment of patellar fractures include anatomic reconstruction of the articular surface, maintenance of the extensor mechanism, and stable fixation that permits early movement [1].

Standard tension-band wiring is a reliable treatment option for simple transverse fractures. However, in the presence of comminution, the dynamic compression achieved through converted tensile forces becomes ineffective, increasing the risk of fixation failure due to instability [2, 3]. The resulting mechanical instability can lead to loss of reduction and the development of posttraumatic osteoarthritis, ultimately causing unsatisfactory long-term outcomes [4]. Therefore, in cases of comminuted fractures, augmentation with screws, plates, or cerclage wire fixation is often performed to enhance stability and facilitate reconstruction.

Given the limitations of conventional treatments, patellar plating constructs have recently emerged as a popular operative method, demonstrating favorable outcomes with few complications [5–12]. Several studies have suggested the superior biomechanical stability of plate osteosynthesis compared to other methods, allowing an early active range of motion [12–14]. A variety of techniques for patellar fixation have been reported, including anterior tension plates with 2.0 mm, 2.7 mm, or 3.5 mm locking or non-locking holes and lateral plates, as well as anatomically designed plates.

However, there is limited evidence regarding the application of plating techniques for multifragmentary patellar fractures, which pose significant challenges in restoring articular fragments and achieving stable fixation [6]. Further advancements in fixation techniques and robust validation of clinical outcomes are essential.

In addition, a recent patellar fracture mapping study confirmed that multifragmentary patellar fractures (AO/OTA 34-C3) tend to have primary horizontal, secondary horizontal, secondary vertical, and main coronal fracture lines that create coronal split, inferior pole, or satellite fragments [15]. Analysis of fracture patterns revealed that comminuted patellar fractures, although complex, can be converted into relatively simple fracture patterns through reduction plating with miniplates. Consequently, we adopted fracture-pattern-driven plate osteosynthesis using multiple miniplates as a treatment strategy for comminuted patellar fractures.

Thus, the purposes of this study were (1) to introduce a fracture pattern-driven patellar plate osteosynthesis technique using multiple miniplates and (2) to evaluate its clinical and functional outcomes. We hypothesized that this novel technique would provide stable fixation, improve fracture reduction, and enable earlier mobilization, leading to favorable outcomes.

## Patients and methods

### Ethical approval

All experimental protocols used in this study were approved by our Institutional Review Board. All procedures and methods performed in studies involving human participants were in accordance with the ethical standards of the institution or practice in which the studies were conducted (IRB numbers: 2019GR0364 and AJOUIRB-DB-2024-611).

### Study population and method

This study was a retrospective review of a prospectively collected cohort and was approved by our institutional review board. The inclusion criteria were as follows: (i) patients diagnosed with comminuted fracture of the patella, including AO/OTA 34-C2 or C3; (ii) operatively treated comminuted patellar fracture with at least 3 mm of displacement in the coronal and sagittal planes; (iii) patients aged over 18 years at the time of injury; (iv) patients with a minimum of 1-year follow-up; (v) patients with pre- and postoperative radiographic evaluations, including computed tomography (CT); and (vi) patients with documented functional scores and Cybex test results. The exclusion criteria were as follows: (i) pathologic fracture; (ii) pre-existing functional limitations of the knee joint; (iii) non-comminuted transverse fractures (AO/OTA 34-C1) that were best managed with tension band construct using K-wire, screws, or a single plate; and (iv) isolated extra-articular inferior pole fractures (AO/OTA 34-A1).

From January 2018 to December 2023, 66 patients were diagnosed with a comminuted fracture of the patella based on preoperative computed tomography and were treated with the fracture-pattern-driven plate osteosynthesis technique using multiple miniplates at two university medical centers. Among them, four patients did not meet the criteria for a minimum 1-year follow-up. A total of 62 patients (34 males and 28 females) were enrolled in the current study.

**Fig. 1:**
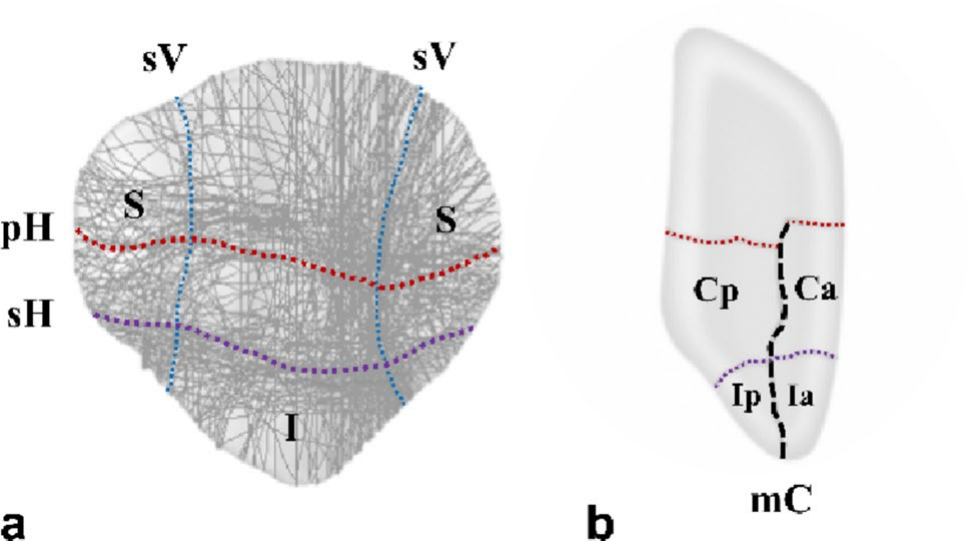
The primary horizontal fracture lines (red dotted lines), secondary horizontal fracture lines (purple dotted lines), and medial and lateral secondary vertical fracture lines (blue dotted lines) were identified as major fracture lines. **a** The major fracture lines from the previous study are as follows: primary horizontal fracture lines (red dotted line), secondary horizontal fracture lines (purple dotted line), and medial and lateral secondary vertical fracture lines (blue dotted line). **b** The main coronal fracture line (black dotted line) and horizontal fracture line produce coronal split fragments by linking. pH primary Horizontal, sH secondary Horizontal, sV secondary Vertical, S Satellite, I Inferior, mC main Coronal, Ca Coronal anterior, Cp Coronal posterior, Ia Inferior anterior, Ip Inferior posterior

The mean patient age was 52.8 ± 13.6 years (range: 19–73). The mechanisms of injury included slipping (39 patients), falling (15 patients), and motor vehicle accidents (eight patients) (Table 1). All patients were preoperatively evaluated using CT to identify comminution of the patella and the presence of free articular fragments. Patients with isolated extra-articular inferior pole fractures (AO/OTA 34-A1) were excluded. All the patients presented with AO/OTA 34-C2 or C3 fractures (C2: 9, C3: 53). The average number of comminuted fragments, defined as those with a diameter greater than 10 mm and displacement exceeding 2 mm, separated by sagittal or coronal plane fracture lines, was 4.5 ± 1.1 (range: 3–8). According to previous studies, fracture patterns were analyzed, including primary horizontal(the largest displaced, typically located between the middle and inferior thirds of the articular surface), secondary horizontal(located along the inferior boundary of the articular facet), secondary vertical(longitudinal and located at the periphery of the medial or lateral facets), and main coronal (connecting the primary and secondary horizontal lines, typically located in the anterior third of the patella) fracture lines, which resulted in coronal splits, inferior pole fragments, or satellite fragments(peripheral fragments separated from the main body by secondary vertical fracture lines) [15]. (Fig. 1). The most common fracture lines were the primary horizontal (96.8%, 60/62) and secondary vertical (98.4%, 61/62). Coronal fragments were identified in 47 patients (75.8%, 47/62), with 32 presenting with isolated coronal split fragments and 15 presenting with impacted coronal split fragments. Satellite fragments were present in all patients and were distributed bilaterally (38.7%, 24/62), laterally (32.3%, 20/62), and medially (29.0%, 18/62). Inferior pole involvement was observed in 50 patients (80.6%, 50/62), including 17 with solitary fragments and 33 with comminution (Table 2). Intraoperative data, including the total operative time, were collected from medical records and fluoroscopic imaging using a picture archiving and communication system (PACS).

**Table 1:** Patient demographics

Variable	*N* (%)
Total number	62
Age (years)	
Mean ± SD(Range)	52.8 ± 13.6 (19–73)
Sex	
Male	34 (54.8)
Female	28 (45.2)
BMI	
Mean ± SD(Range)	23.9 ± 2.5 (18.6–29.9)
ASA classification	
I	39 (62.9)
II	15 (24.2)
III	8 (12.9)
Mechanism	
Slip down	38 (61.3)
Fall down	16 (25.8)
Motor vehicle accident	8 (12.9)
Laterality	
Right	30 (48.4)
Left	32 (51.6)

**Table 2:** Fracture characteristics

Variable	AO/OTA 34-C2 (*n* = 9)	AO/OTA 34-C3 (*n* = 53)	Total (*n* = 62)
Open fracture, N(%)	0(0)	4(7.5)	4(6.5)
Numbers of fragments			
Mean ± SD(Range)	3.0 ± 0.7(3–5)	4.5 ± 1.1(3–8)	4.5 ± 1.1 (3–8)
Fracture line, N(%)			
Primary Horizontal	9(100)	51(96.3)	60(96.8)
Primary Vertical	0(0)	7(13.2)	7(11.3)
Secondary Horizontal	3(33.3)	49(92.5)	52(83.9)
Secondary Vertical	9(100)	52(98.1)	61(98.4)
Presence of coronal fragment, N(%)			
None	6(66.6)	9(17.0)	15 (24.2)
Isolated	3(33.3)	29(54.7)	32 (51.6)
Impacted	0(0)	15(28.3)	15(24.2)
Satellite fragment, N(%)			
None	0(0)	0(0)	0(0)
Medial	4(44.4)	14(26.4)	18(29.0)
Lateral	5(55.6)	15(28.3)	20(32.3)
Both	0(0)	24(45.3)	24(38.7)
Inferior pole involvement, N(%)			
None	5(55.6)	7(13.2)	12(19.4)
Intact	2(22.2)	15(28.3)	17(27.4)
Comminution	2(22.2)	31(58.5)	33(53.2)

### Surgical technique

#### Surgical approach

A longitudinal midline incision approximately 10 cm in length was made on the anterior aspect of the knee, from the lower half of the quadriceps tendon to the upper half of the patellar tendon. Full-thickness flaps deep at the fascial level were created to minimize soft tissue complications. Although torn at the primary fracture line, the retinaculum attached to fragments is preserved. The anterior cortex and articular surface are inspected for concealed comminution and free fragments.

#### Fixation technique

**Fig. 2:**
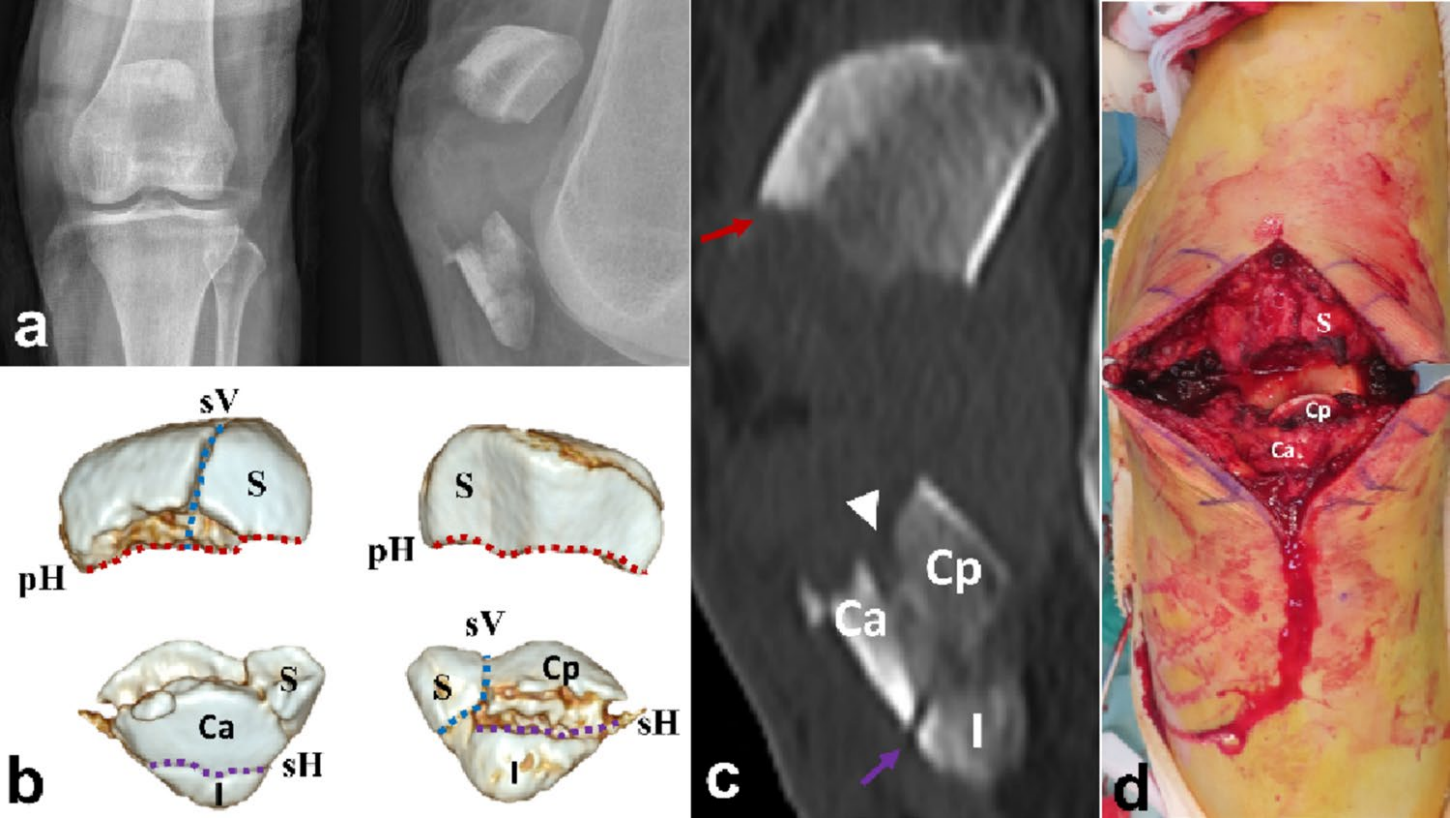
**a** A 62-year-old female patient diagnosed with AO/OTA 34-C3 associated with coronal split fractures. **b** Fracture lines and fragments were indicated based on the patellar fracture analysis. **c** The primary horizontal fracture line is located on the middle level of the patella (red arrow), and the secondary fracture line is located on the lower boundary of the patella (purple arrow). The main coronal fracture line (arrowhead) separated the anterior and posterior coronal fragments. **d** Intraoperatively, those coronal fragments were split. pH primary Horizontal, sH secondary Horizontal, sV secondary Vertical, S Satellite, I Inferior, mC main Coronal, Ca Coronal anterior, Cp Coronal posterior

The primary fracture line was identified and debrided. The main fracture gap served as a window for coronal fragment reduction (Fig. 2) and palpation of articular step-off. The guiding principles of this technique are as follows: first, to simplify comminuted fracture patterns using miniplates or screws to neutralize secondary vertical fracture lines or the main coronal fracture line; and second, to apply anterior tension-band plating across the primary horizontal fracture line while simultaneously stabilizing the secondary horizontal fracture line. The miniplates and screws were derived from the 1.5 mm, 2.0 mm, and 2.4 mm Compact Hand Set (DePuy Synthes, USA) or the 1.5 mm and 2.0 mm ARIX Hand System (Jeil Medical, Republic of Korea), both originally designed for the management of hand fractures.

### The sequence of procedure

#### Conversion to simple fracture by managing the satellite and coronal split fragments

**Fig. 3:**
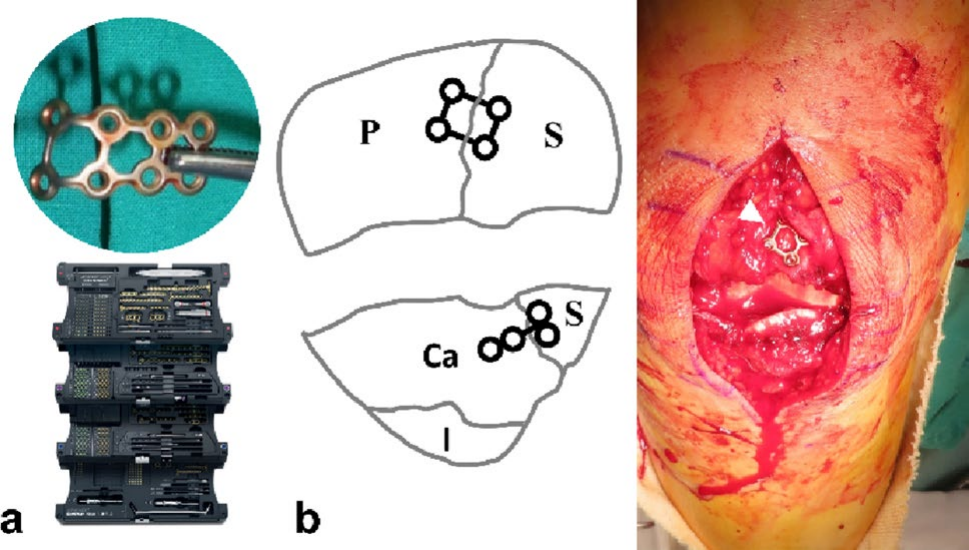
**a** The 1.5 mm strut-type mini plate from the 1.5 mm Compact Hand Set (DePuy Synthes, USA) was slightly over-bent before application. **b** The satellite proximal and distal fragments were stabilized with anterior 1.5 mm strut-type (arrowhead) and T-shape mini plates. P proximal, S satellite, Ca Coronal anterior, I inferior

The Satellite proximal fragments created by the secondary vertical fracture line were reduced to the main fragment and stabilized with a 2.0 mm lag screw or a 1.5 mm strut-type mini plate. The plate was slightly overbent to prevent cortical widening, with screws inserted to subchondral depth (Fig. 3).

**Fig. 4:**
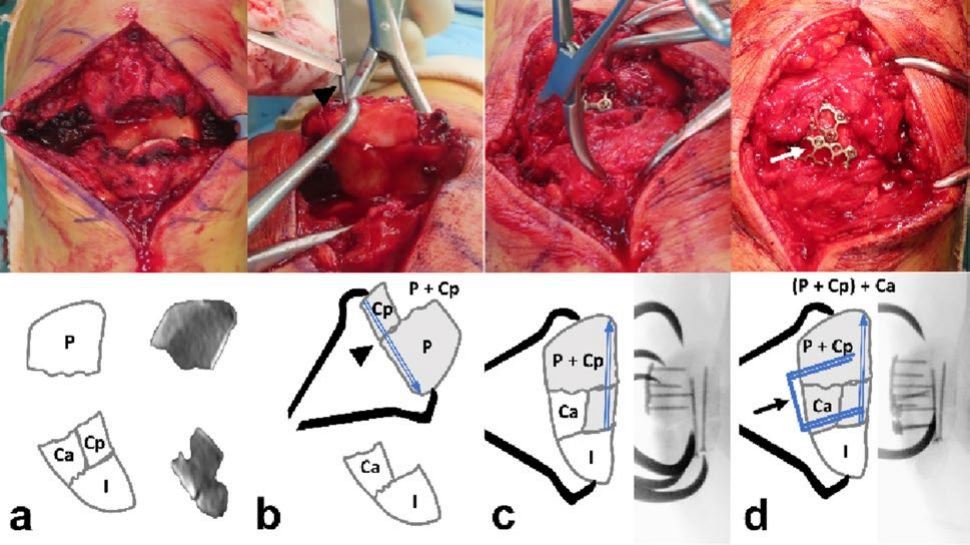
**a** The primary horizontal fracture was widely displaced. **b** After taking out the posterior coronal free articular fragment from the distal fracture segment, it was reduced and fixed with two 1.5 mm embedded screws(arrowhead) using the backward flip approach. **c** Reduction of primary horizontal fracture using pointed reduction clamp (**d**) Stabilization by anterior 1.5 mm strut-type reduction plate (black and white arrow). P proximal, Ca Coronal anterior, Cp Coronal posterior, I inferior

**Fig. 5:**
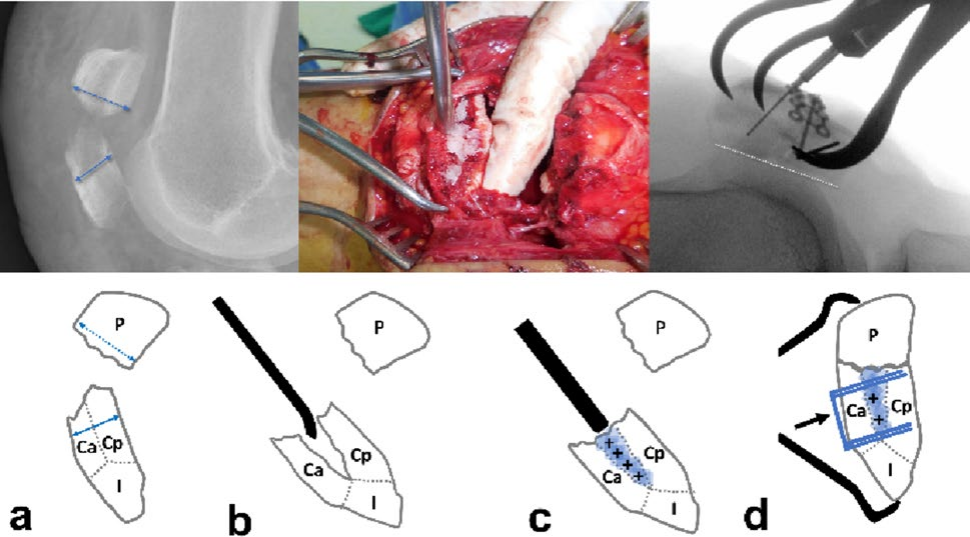
**a** A 57-year-old female patient diagnosed with AO/OTA 34-C3 associated with coronal split fractures with impaction. The width of distal fragments (dotted blue line) was decreased compared with that of proximal fragments. (blue line) (**b**) Dis-impaction using curved mosquito clamp or osteotome. **c** Morselized chip bone graft (**d**) Stabilization with Anterior to posterior direction screws afterward through the 1.5 mm anterior reduction plate (black arrow). P proximal, Ca Coronal anterior, Cp Coronal posterior, I inferior, **+** Morselized chip bone

The Coronal split fragments present as free articular or impacted types. Free fragments are removed from the distal segment. The proximal segment is flipped longitudinally to allow reduction through the fracture window, where the fragment is stabilized under interfragmentary compression using a pointed reduction clamp and fixed with at least two 1.5 mm embedded subchondral screws (Fig. 4). Conversely, the impacted fragment, which retained an intact distal subchondral cortical hinge, was disimpacted through the fractured window using a mosquito clamp while preserving the intact hinge at the distal part. The defect is filled with morselized chip bone graft. The reduction is stabilized by anterior-to-posterior screws through a 1.5 mm anterior reduction plate bridging the primary horizontal fracture (Fig. 5).

#### Reduction of horizontal fracture lines

Following fixation of the satellite fragment or coronal articular fragment, the main transverse fracture formed by the primary and secondary horizontal fracture lines remained. The fracture line is reduced using a pointed reduction clamp. A small split incision was made along the patellar tendon to allow advancement of the clamp. One prong of the clamp was positioned to encircle the inferior pole fragment, whereas the other was placed on the proximal fragment. The reduction was provisionally fixed with K-wires or by 1.5 mm anterior reduction plating.

#### Patellar tension-band plating

**Fig. 6:**
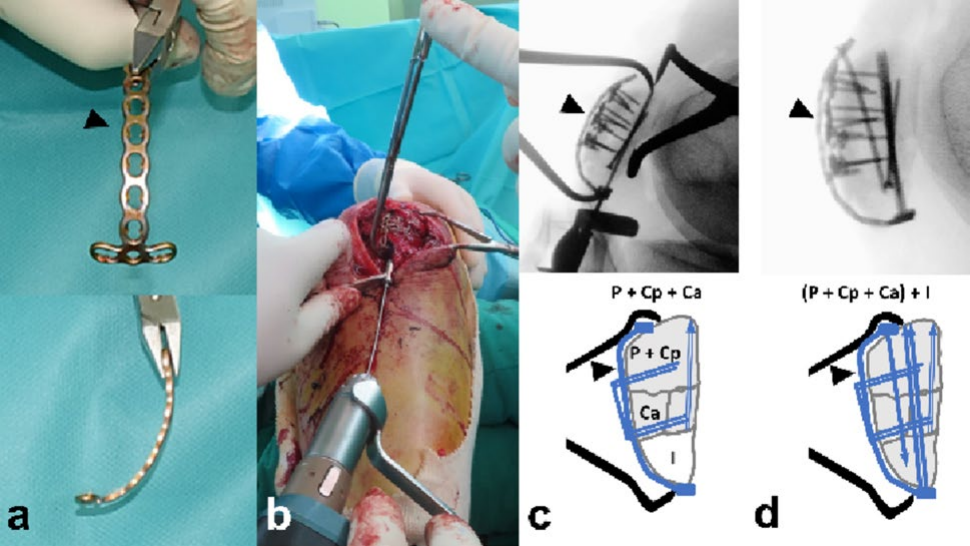
**a** The 2.4 mm (Compact Hand Set; DePuy Synthes, USA) or 2.0 mm (Arix Hand System; Jeil Medical, Republic of Korea) T-type plate (arrow head) was slightly overbent. **b** After creating a 2.0 cm incision in the patellar tendon, the plate was advanced beneath the tendon, positioning the three-holed head of the plate under the inferior pole. **c** A ball-tip clamp or Weber clamp was used to press the plate firmly to enhance compression on the inferior pole fragment created by the secondary horizontal fracture line. **d** The insertion of two conventional cortical screws across the primary horizontal fracture line to provide dynamic compression and secure the plate against the anterior patellar cortex. An additional locking screw was then inserted into the proximal fragment

The 2.4 mm–2.0 mm T-type plate was bent along the anterior surface of the reduced patella to overbend it slightly (Fig. 6a). Depending on the size of the patella, the shaft was trimmed and shaped accordingly. After creating a 2.0 cm incision in the patellar tendon, the plate was advanced beneath the tendon, positioning the three-hole head of the plate under the inferior pole (Fig. 6b). A ball-tip clamp or Weber clamp was used to firmly press the plate against the inferior pole, thereby enhancing compression of the inferior pole fragment created by the secondary horizontal fracture line (Fig. 6c). Two conventional cortical screws were inserted across the primary horizontal fracture to provide dynamic compression and secure the plate. An additional locking screw was then inserted into the proximal fragment (Fig. 6d). For stabilization of the primary or secondary horizontal fracture lines, at least two long screws with an average length of 40 mm were inserted retrogradely through the split in the patellar tendon, crossing the primary horizontal fracture line. This anterior plate-and-screw construct functions similarly to the wire and pins used in traditional tension-band wiring in terms of withstanding tensile forces and maintaining compression at the fracture site. Therefore, this method is referred to as **tension-band plating**.

### Postoperative protocols

Rehabilitation typically begins 2–3 days after the surgery. Isometric quadriceps-strengthening exercises, including seated exercises and straight leg raises, were initiated under the guidance of a physical therapist. Following the initial dressing, the knee was supported using a hinged knee brace. Gradual passive range of motion (ROM) exercises were performed using either a continuous passive motion (CPM) device or with the assistance of a physical therapist, with adjustments made according to the receiving hospital’s protocol. The patients were instructed to achieve 90° knee flexion at 4 weeks postoperatively and 120° flexion at 6 weeks. Deep squat exercises were generally performed at 8 weeks. The entire rehabilitation process was monitored and adjusted during the outpatient clinic visits. Partial weight-bearing with crutches was initiated immediately after surgery and maintained for 4 weeks, depending on the patient’s pain tolerance.

### Outcome measurement

Postoperative follow-up visits were scheduled at 2 weeks and then at 1, 2, 3, 6, 9, and 12 months. Standard plain radiographs were routinely obtained. An immediate postoperative CT scan was performed to evaluate the quality of articular surface reduction. The articular reduction was classified as ‘anatomical’ (no step off) with perfect alignment of all fracture fragments., ‘good’ (0–2 mm step-off) with restoration of most of the articular surface and minimal residual deformity, or ‘poor’ (over 2 mm step-off) [16]. Radiologic evidence of bony union was characterized by the restoration of bony trabecular continuity and disappearance of the fracture line.

Functional outcomes were assessed using Cybex tests and Lysholm scores, along with a range of motion at 6 and 12 months. The isokinetic concentric knee extension peak torque was measured before testing using a Cybex II dynamometer calibrated with standard weights. The flexion and extension strengths were measured at an angular velocity of 60°/s. The proportional deficit in peak isokinetic knee extension torque of the injured limb was calculated relative to that of the uninjured limb. Outcomes were categorized as good for deficits below 30%, moderate for deficits between 30% and 44%, and poor for deficits exceeding 45%, according to the criteria established by Levack et al. [4, 17].

As the primary goal of this study was to determine the feasibility of the technique and characterize functional recovery up to 12 months, the assessment of posttraumatic degenerative changes was beyond the objectives of the current analysis. Therefore, long-term radiographic evaluation for osteoarthritis was not performed.

## Results

The fracture pattern-driven plate osteosynthesis technique using multiple miniplates was applied to 62 patients with comminuted patellar fractures classified as AO/OTA 34-C2 (14.5%, 9/62) or 34-C3 (85.5%, 53/62). The final union rate was 100% (62/62 patients). Almost all the patients were treated using the anterior approach (95.2%, 59/62). The average number of reduction plates used was 1.7 ± 0.8 (range: 1–4). The mean length of retrograde screws was 39.9 ± 1.7 mm (range: 36–44 mm). As detailed in Tables 2 and Table 3, the AO/OTA 34-C3 group presented with higher comminution and a greater prevalence of coronal split fragments compared to the AO/OTA 34-C2 group, consequently requiring a higher average number of reduction plates. The mean operation time was 98.0 ± 24.2 minutes (range: 66–132) in the AO/OTA 34-C2, 114.7 ± 30.3 minutes (range: 66–235) in AO/OTA 34-C3, and 112.2 ± 29.9 minutes (range: 66–235) overall. The immediate postoperative articular reduction was classified as ‘anatomic’ in 72.6% of cases (45/62) and ‘good’ in 27.4% of cases (17/62), with no cases classified as ‘poor.’ The average follow-up duration for all patients was 479.3 ± 31.5 days (range: 398–600).

There were five cases of complications, including two major complications requiring unexpected additional surgery. One patient experienced loss of reduction in the inferior pole fragments due to additional slippage and underwent revision fixation surgery (Case No. 36). Another patient with diabetes mellitus developed an acute postoperative infection that was successfully managed with debridement, antibiotics, and implant retention (DAIR), resulting in fracture healing without infection recurrence (Case No. 34). Among the minor complications, one patient experienced loss of reduction due to breakage of the plate (head of a 2.0 mm T-plate), with healing achieved despite mild displacement, which did not result in significant functional impairment (Case No. 49). Additionally, one patient presented with asymptomatic nonunion characterized by mild displacement of the inferior pole fracture (Case No. 2). Only one patient reported mild implant irritation and patellar tendinitis, which were successfully managed with conservative treatment (Case No. 53) (Table 3).

**Table 3:** Surgical details and results

Surgical details	AO/OTA 34-C2 (*n* = 9)	AO/OTA 34-C3 (*n* = 53)	Total (*n* = 62)
Approach, N(%)			
Anterior	9(100)	50(94.3)	59(95.2)
Anterior + Para-patella	0(0)	3(5.1)	3(4.8)
Number of Reduction Plate Mean ± SD(Range)	1.2 ± 0.4 (1–2)	1.8 ± 0.8 (1–4)	1.7 ± 0.8 (1–4)
Length of Retrograde screw			
Mean ± SD(Range)	40.2 ± 1.6(38–42)	39.8 ± 1.7(36–44)	39.9 ± 1.7(36–44)
Operation time(min)			
Mean ± SD(Range)	98.0 ± 24.2 (66–132)	114.7 ± 30.3 (66–235)	112.2 ± 29.9(66–235)
**Results**			
Union, N(%)	9(100)	53(100)	62(100)
Quality of reduction			
Anatomical	7(77.8)	38(71.7)	45(72.6)
Good	2(22.2)	15(28.3)	17(27.4)
Poor	0(0)	0(0)	0(0)
Follow-up duration(day)			
Mean ± SD(Range)	469.6 ± 28.7(431–512)	478.2 ± 32.1(398–600)	479.3 ± 31.5(398–600)
Complications, N(%)			
None	8(88.9)	49(92.5)	57(91.9)
Implant irritation	0(0)	1(1.9)	1(1.6)
Infection	0(0)	1(1.9)	1(1.6)
Reduction loss	1(11.1)	1(1.9)	1(1.6)
Plate breakage	0(0)	1(1.9)	1(1.6)
Additional surgery	1(11.1)	1(1.9)	2(3.2)

**Fig. 7:**
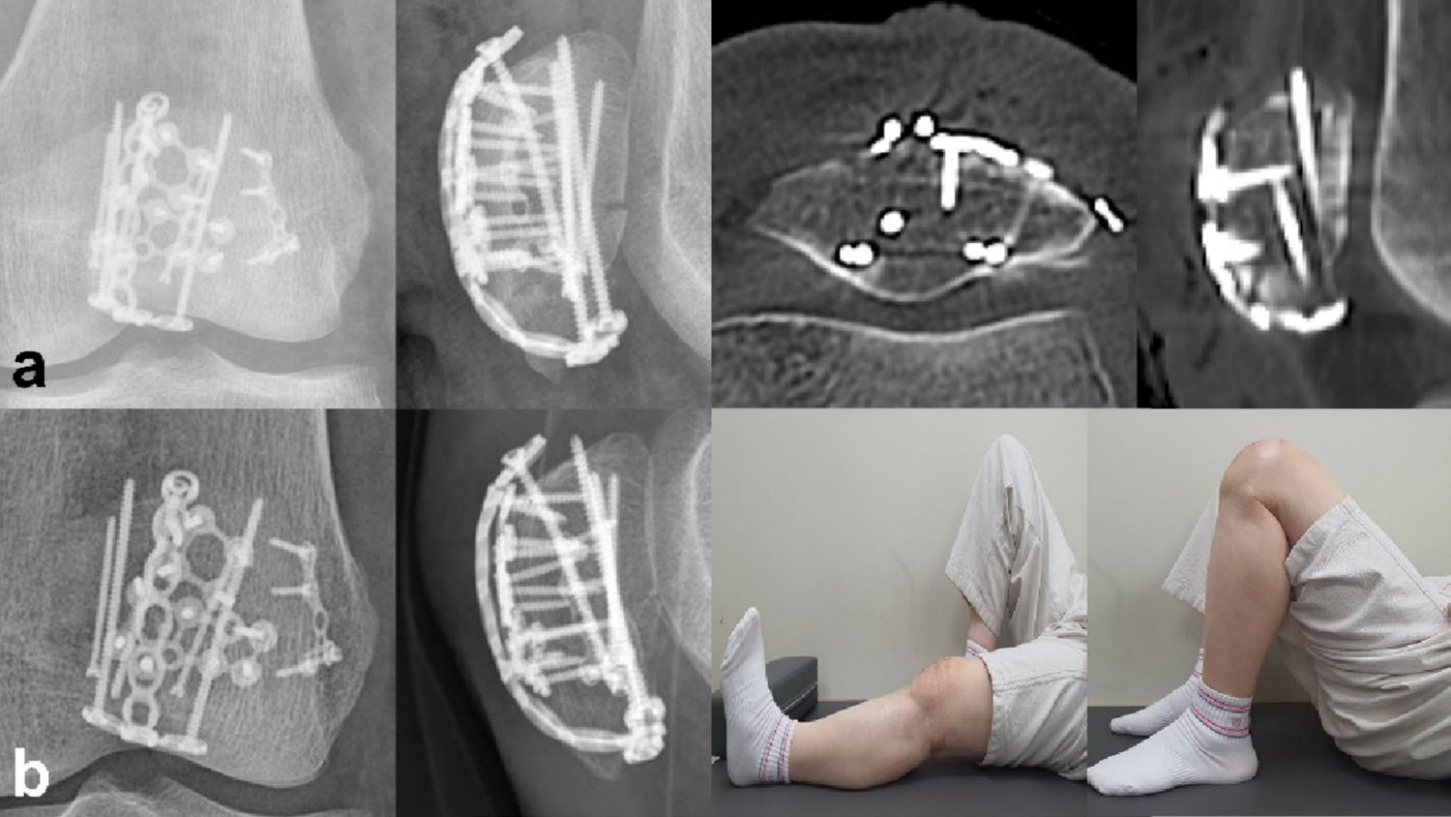
**a** Postoperative radiographs show the tension-band plating with multiple mini plates. All identified fragments were anatomically stabilized. There was no articular step-off showing anatomical reduction. **b** After 6 months, the radiologic bony union was achieved, and the range of motion was fully recovered

The average final range of motion was 132.9 ± 6.8 degrees (range: 120–140). The average Lysholm knee scores at 6 and 12 months postoperatively were 70.3 ± 10.0 (range: 45–90) and 89.1 ± 6.7 (range: 73–98), respectively. At the final follow-up, 32.3% (20/62) of the patients demonstrated excellent function, whereas 43.5% (27/62) showed good function (Fig. 7).

The average isokinetic peak torque of the knee extensor muscles was significantly lower in the injured leg than in the uninjured leg at 6 and 12 months postoperatively. The isokinetic peak torque deficits, indicating the percentage strength loss of the injured limb compared to the uninjured limb, were 49.2 ± 12.4% (range: 22–80) and 24.6 ± 13.0% (range: 3–58), respectively. According to the criteria from Levack et al. [4], good and moderate quadriceps strength was observed in 66.7% (42/62) and 24.2% (15/62) of patients, respectively. Furthermore, the body mass–normalized peak extension torque (Nm/kg) was significantly lower in the injured limb than in the uninjured limb at both time points. (Table 4).

**Table 4:** Clinical outcomes with Lysholm score, isokinetic concentric knee extension peak torque deficit, and normalized extension peak torque at an angular velocity of 60°/s (Cybex test)

Variables	AO/OTA 34-C2 (*n* = 9)	AO/OTA 34-C3 (*n* = 53)	Total (*n* = 62)
Final ROM			
Mean ± SD(range)	131.7 ± 7.9 (120–140)	133.1 ± 6.7 (120–140)	132.9 ± 6.8(120–140)
*Lysholm score*			
6 months			
Mean ± SD(range)	68.8 ± 11.5(48–85)	70.6 ± 9.9(45–90)	70.3 ± 10.0(45–90)
12 months			
Mean ± SD(range)	90.7 ± 5.7(80–98)	88.9 ± 6.8 (73–98)	89.1 ± 6.7(73–98)
*Extension peak torque deficit (%)*			
6 months			
Mean ± SD(range)	50.6 ± 15.3(28–78)	49.0 ± 12.0(22–80)	49.2 ± 12.4(22–80)
12 months			
Mean ± SD(range)	23.4 ± 8.7(10–36)	24.8 ± 13.6 (3–58)	24.6 ± 13.0(3–58)
*Normalized extension peak torque (Nm/kg)*			
6 months			
*Uninjured*			
Mean ± SD(range)	1.39 ± 0.43(0.72–2.45)	1.38 ± 0.44(0.69–2.59)	1.38 ± 0.44(0.69–2.59)
*Injured*			
Mean ± SD(range)	0.77 ± 0.38(0.21–1.88)	0.77 ± 0.39(0.25–1.91)	0.77 ± 0.39(0.21–1.91)
12 months			
*Uninjured*			
Mean ± SD(range)	1.52 ± 0.36(1.29–2.38)	1.55 ± 0.39(1.07–2.59)	1.55 ± 0.39(1.07–2.59)
*Injured*			
Mean ± SD(range)	0.98 ± 0.39(0.21–1.75)	0.99 ± 0.41(0.26–1.97)	0.99 ± 0.40(0.21–1.97)

## Discussion

In this retrospective study, a fracture pattern–driven plate osteosynthesis technique using multiple miniplates was applied to 62 patients with comminuted patellar fractures, most of whom were classified as AO/OTA 34-C3. The technique demonstrated excellent postoperative outcomes, with 72.6% of the patients achieving anatomical reduction and the remaining 27.4% classified as good. None of the patients were classified as poor, and the final union rate was 100%. The average postoperative range of motion was 132.9°, reflecting satisfactory restoration of joint mobility. Clinical outcomes were favorable throughout the follow-up period, and the overall complication rate was low, with only two patients requiring unplanned reoperation. Moreover, postoperative functional outcomes, particularly the Lysholm scores and quadriceps strength, showed marked improvements between 6 and 12 months postoperatively. These improvements were supported by isokinetic testing, which demonstrated recovery of extensor muscle function over time. More than 75% of the patients achieved good-to-excellent function at the final follow-up, highlighting the effectiveness of this novel technique not only in achieving radiographic healing but also in facilitating meaningful functional recovery.

Surgical management of comminuted patellar fractures, particularly the AO/OTA 34-C type, remains challenging. Although tension-band wiring remains the most commonly used technique, it is associated with high complication and reoperation rates, particularly in cases involving severe comminution or fractures of the inferior pole. Previous studies have reported an increased risk of reduction loss and implant failure owing to insufficient mechanical stability. Additionally, soft tissue complications are common and often result from wire breakage or migration [4, 10, 18].

Biomechanical studies have consistently demonstrated that anterior or anterolateral locking plate constructs provide superior stability compared with tension-band wiring [12, 19–21]. Specifically, these investigations found that fixed- and variable-angle plating significantly reduced interfragmentary displacement and rotational instability while increasing resistance to mechanical failure under cyclic loading, regardless of fracture complexity. Furthermore, existing literature [5, 6, 19–27] supports favorable clinical outcomes associated with plate fixation systems for patellar fractures. These include high radiographic union rates, satisfactory restoration of the knee range of motion, and improved functional scores. Moreover, the incidence of major and minor complications was relatively low (Table 5). Recent meta-analyses [8, 10] further support these findings, reinforcing the clinical efficacy and safety of plate-based fixation techniques in the management of patellar fractures.

**Table 5:** Plate osteosynthesis of AO/OTA 43-C from the previously published literature

Year	Author	Study design	Position of plate	Type of plate	Implant	Number of patients	Outcome		Union rate	Complications
							ROM	Functional score		
Our study		Retrospective	Anterior	Non-anatomical, Locking	2.0/2.4 mm plate 2.0/2.4/2.7 Screw	62	132.9 ± 6.8(120–140)	89.1 ± 6.7(73–98)	100%	SSI: 1 Reduction loss: 1 Metal failure: 1 Irritation: 1
2014 [22]	Talor et al.	Prospective	Anterior	Non-anatomical, Locking	2.4/2.7 mm X-plate, Mesh plate	8	0-129	NS	100%	None
2015 [23]	Lorich et al.	Retrospective	Multiplanar	Non-anatomical, Locking	2.4/2.7 mm mesh plate	9	Full extension to 143	KOS-ADLS 84, LEFS 74, SF-36 PCS 50, SF-36 MCS 56	100%	HR for HI: 1
2016 [24]	Wild et al.	Prospective	Multiplanar	Anatomical, Locking	3.5 mm variable angle-stable bilateral patellar plate	19	125	125 ± 7.8(80–145)	100%	HR for SSI:1 Reduction loss:1
2017 [25]	Singer et al.	Prospective	Anterior	Non-anatomical, Non-locking	Mesh plate, low profile 1.5 mm titanium	9	128.9 ± 6.3(0-140	Böstman scale: 27.2 ± 3.1(22–30) Lysholm: 89.1 ± 4.9(82–95)	100%	SSI: 1 DVT: 1
2018 [26]	Moore et al.	Retrospective	Anterior	Non-anatomical, Locking	2.4/2.7 mm variable angle X-plate, 2.4/2.7 mm locking plate	20	averaged 1-126 (median: extension = 0, flexion = 130	KOS-ADLS:57.2(20–74) LEFS:58.9(15–80)	100%	HI:4 HR for SSI:1 Fixation Failure:1
2019 [27]	Ellwein et al.	Prospective	Anterior	Anatomical, Locking	Patella ArrowPlate, StarPlate	19 (Total 20, A1: 1)	143	Tegner: 4.1 Lysholm: 97 Kujala: 97	95%	Reduction loss:1 Reactive bursitis:1 Renewed fracture:1 HR:4
2021 [28]	Meng et al.	Retrospective	Anterior	Non-anatomical, Locking	2.7 mm variable angle-locking plate	40	124 ± 11	Lysholm:90.2 ± 3.9	100%	HR:4, Knee contracture: 1, PTOA: 1
2021 [5]	Shymon et al.	Prospective	Anterior	Non-anatomical, Locking	2.4/2.7 mm locking plate	18	131 ± 7	SF-36 PCS:55.7 ± 36.9 SF-36 MCS:58.9 ± 33.4	100%	Implant irritation:5
2022 [6]	Buschbeck et al.	Retrospective	Anterior	Anatomical, Locking	Patella SuturePlate, ArrowPlate, StarPlate	29	131(100–150)	Lysholm: 84.7(45–100) Tegner score 4.3(3–10)	100%	HR for IR:7
2024 [29]	Bickel et al.	Retrospective	NS	NS	NS	23	Flexion lag:0(0.0) Extension lag:1.67(5.77)	Lysholm:89.8 ± 11.9	100%	HR for SSI:2
2025 [32]	Shaath et al.	Retrospective	Anterior	Anatomical, Locking	2.7 mm variable angle-locking plate	58(Total 61, A1:2,B1:1)	110(90–130)	NS	89%	Infection:2 Arthrobibrosis:3 Fixation Failure:2

KOS-ADLS Knee Outcome Survey Activities of Daily Living Scale, LEFS Lower Extremity Functional Scale, SF-36 PCS Short Form Survey Instrument 36 Physical Component Summary, SF-36 MCS Short Form Survey Instrument 36 Mental Component Summary, HR Hardware Removal, HI Hardware Irritation, SSI Surgical Site Infection, DVT Deep Vein Thrombosis, PTOA Post-Traumatic OsteoArthritis, NS Not Specified

**Fig. 8:**
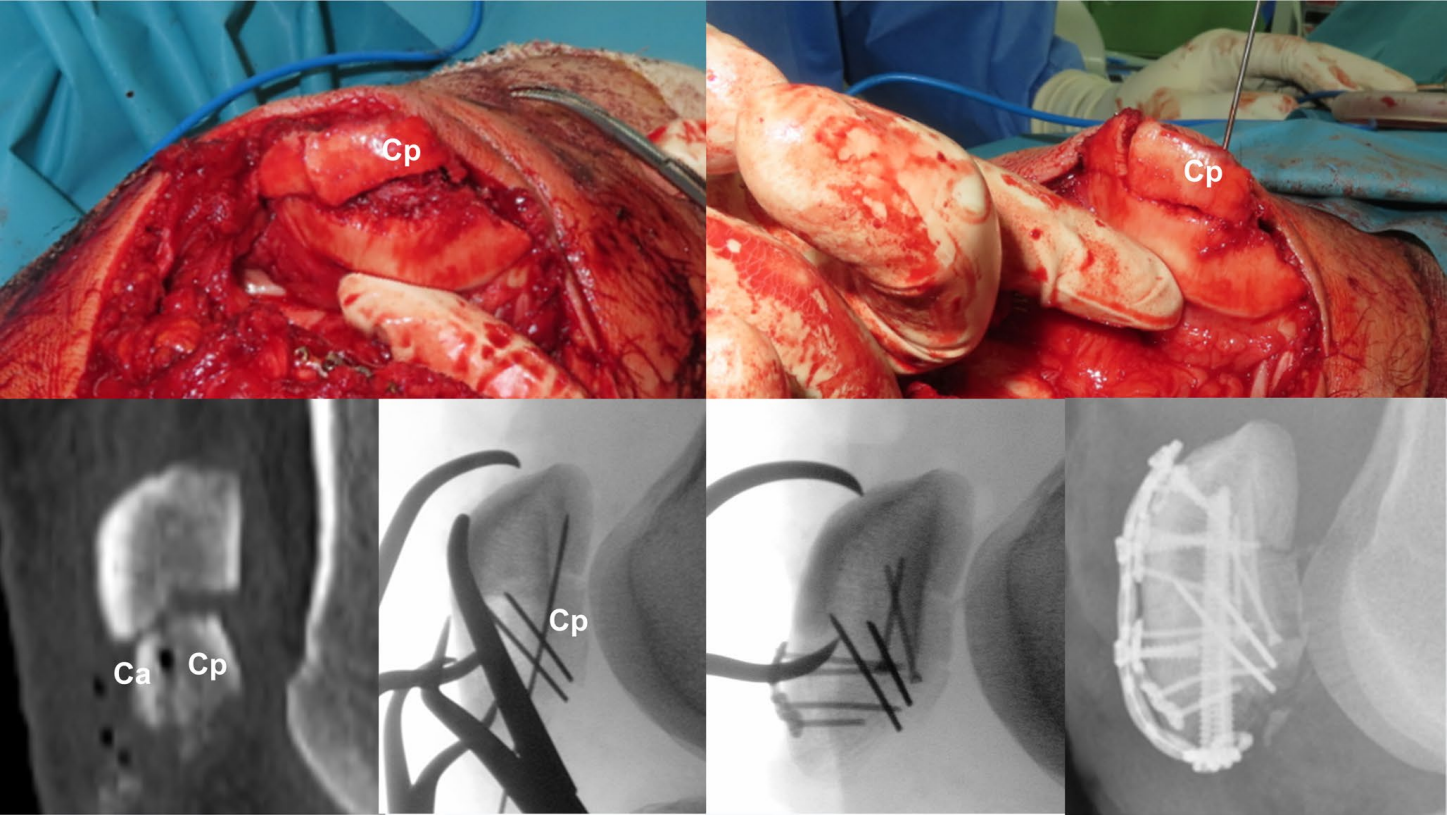
A 52-year-old male patient diagnosed with AO/OTA 34-C3 associated with anterior and posterior coronal split fractures in the distal fragment. Two comminuted posterior coronal fragments were reduced and fixed with two 1.5mm embedded screws and two 1.4mm K-wires on the proximal fragment through a forward flipping technique. Cp coronal posterior Ca coronal anterior

The key advantage of this novel technique is its adaptability to a wide range of fracture morphologies. Stable and fragment-specific fixation can be achieved even in highly comminuted fractures by utilizing a small anterior strut-type miniplate or an embedded screw. This individualized approach resulted in favorable radiological and clinical outcomes despite the technical challenges posed by complex fragmentation. Moreover, each surgical step was thoroughly described and supported by illustrations that provided clear guidance on the technical aspects of the procedure. We strongly propose that multifragmentary fractures be reconstructed sequentially, beginning with the reduction in the satellite or coronal fragments. This strategy facilitates simplification of the fracture geometry, allows for a more accurate assessment of anatomical alignment, and minimizes the risk of reduction loss, in contrast to approaches that prioritize the reduction of the main fragments from the outset. In particular, we emphasize a novel approach for managing free articular-type coronal split fragments, which often require removal from the distal fracture segment for proper handling. The proximal articular fragment was carefully flipped along its longitudinal axis to allow direct reduction and fixation of the intercalary coronal fragments. This “forward flipping technique” provides full visualization of the articular surface and enables precise reduction tailored to the specific local anatomy of the fracture (Fig. 8).

This study distinguishes itself by incorporating objective postoperative strength testing, moving beyond reliance on subjective scores and range of motion. Using isokinetic dynamometry, we quantified quadriceps function via isokinetic peak torque and body mass–normalized extension torque at 6 and 12 months. Although strength improved longitudinally, clinically significant deficits persisted relative to the uninjured limb. At 12 months, the isokinetic peak torque deficit remained 24.6 ± 13.0% (range, 358), and the body mass–normalized peak extension torque averaged 0.99 ± 0.40 Nm/kg (range, 0.21–1.97) despite appropriate rehabilitation. These findings quantify residual functional impairment often missed by subjective scores, supporting the necessity for rehabilitation beyond 12 months and validating the importance of strength-based monitoring in fracture pattern-driven osteosynthesis.

The average of body mass-normalized extension torque of the uninjured limb at 6 and 12 months was 1.38 ± 0.44 (Nm/kg, range: 0.69–2.59) and 1.55 ± 0.39 Nm/kg (range: 1.07–2.59), respectively. Previous studies [30, 31] have reported that factors such as age, sex, athletic status, and type of sport can influence muscle strength values. In our cohort, the male-to-female ratio was approximately 1:1, with a mean age of 52.8 ± 13.6 years, which may partially explain the relatively lower values compared to normative data reported in previous studies.

The current study has several limitations. First, the study had a retrospective design with no control or comparison groups. Establishing a comparator cohort treated with conventional tension-band wiring was not clinically feasible, as previous studies have established that traditional techniques often fail to provide sufficient mechanical stability for the complex AO/OTA 34-C2 and C3 fracture patterns predominant in our cohort. Consequently, the primary aim of this study was to demonstrate the feasibility of this novel fracture pattern-driven technique and to characterize its clinical and functional outcomes, rather than to assess comparative effectiveness against methods known to yield inferior results in comminuted fractures. Despite the absence of a control group, this study provides valuable insights by analyzing a relatively large number of cases and the highest number of C3 fractures. Second, the fixation construct was not unified and differed according to the fracture pattern, which may have introduced variability in the outcomes. A previous fracture-mapping study identified several representative fracture patterns. However, the number and location of the fragments varied along the spectrum, indicating that a single standardized implant is not feasible. Therefore, fracture-pattern-specific plate osteosynthesis is necessary. Third, posttraumatic degenerative joint changes were not assessed in this study, although they represent a significant long-term complication. The omission of posttraumatic degenerative joint assessment is particularly relevant for AO/OTA type 43-C fractures, which have a known tendency to progress to posttraumatic osteoarthritis during long-term follow-up. However, the current study was not long-term, which further limited the assessment of posttraumatic degenerative joint changes. Furthermore, the quality of reduction and the extent of cartilage injury are important factors influencing this degenerative process. In our cohort, the immediate postoperative articular reduction was classified as ‘anatomic’ in 72.6% and ‘good’ in 27.4% of cases, with no cases rated as ‘poor.’ However, because the extent of the initial cartilage damage was not thoroughly documented in all patients, its impact on long-term outcomes could not be evaluated. Nevertheless, the authors believe that the superiority in the quality of reduction in our cohort can guarantee long-term outcomes, including posttraumatic osteoarthritis. Fourth, the cost-effectiveness of the fixation method was not evaluated. In our clinical setting, the wiring system is significantly less expensive than locking plate constructs. Although multiple small plates were used in this study, resulting in a relatively high total cost, the overall cost remained lower than that of commercially available anatomical patellar plates. Further investigation into the cost-effectiveness of our novel fixation strategy is needed to facilitate its general acceptance in clinical practice.

## Conclusion

In conclusion, the fracture pattern-driven plate osteosynthesis technique demonstrated excellent radiological healing, favorable functional outcomes, and low complication rates in comminuted patellar fractures. This novel technique offers secure fragment-specific fixation, even in highly comminuted fractures. These findings suggest that it is a promising alternative to conventional fixation and warrants further validation in long-term cost-effectiveness studies.

## Data Availability

No datasets were generated or analysed during the current study.
